# Clinical Evaluation of a Novel Urine Collection Kit Using Filter Paper in Neonates: An Observational Study

**DOI:** 10.3390/children8070561

**Published:** 2021-06-29

**Authors:** Nobuhiko Nagano, Takayuki Imaizumi, Takuya Akimoto, Midori Hijikata, Ryoji Aoki, Ayako Seimiya, Aya Okahashi, Kaori Kawakami, Atsushi Komatsu, Kei Kawana, Ichiro Morioka

**Affiliations:** 1 Department of Pediatrics and Child Health, Nihon University School of Medicine, 30-1, Oyaguchi-kamimachi, Itabashi-ku, Tokyo 1738610, Japan; nagano.nobuhiko@nihon-u.ac.jp (N.N.); imaizumi.takayuki@nihon-u.ac.jp (T.I.); akimoto.takuya@nihon-u.ac.jp (T.A.); hijikata.midori@nihon-u.ac.jp (M.H.); aoki.ryoji@nihon-u.ac.jp (R.A.); seimiya.ayako78@nihon-u.ac.jp (A.S.); okahashi.aya@nihon-u.ac.jp (A.O.); 2 Department of Obstetrics and Gynecology, Nihon University School of Medicine, 30-1, Oyaguchi-kamimachi, Itabashi-ku, Tokyo 1738610, Japan; kawakami.kaori@nihon-u.ac.jp (K.K.); komatsu.atsushi@nihon-u.ac.jp (A.K.); kawana.kei@nihon-u.ac.jp (K.K.)

**Keywords:** dermatitis, filter paper, neonate, stool, urine bag

## Abstract

Urine bags are commonly used to collect urine samples from neonates. However, the sample can be contaminated by stool, or detachment of the bag due to body movement can lead to failure of the collection. A qualitative urine collection kit containing ten filter papers of 3.2 mm diameter was developed and clinically verified among 138 neonates. During a single diaper change (approximately 3 h), the rate of urine collection was calculated. Urine collection was considered to be successful if any filter paper in the urine collection sheet turned from blue to white. Of the 127 neonates who passed urine, 122 had a change in the filter paper. The urine collection rate was 96%, with changes in all 10 filter papers observed in 98 neonates (80%). Urine collection rate was not influenced by sex (*p* = 1.00), age at collection (*p* = 0.72), preterm birth (*p* = 1.00), low birth weight (*p* = 0.92), or fecal contamination (*p* = 1.00). The incidence of dermatitis was not higher than in the group in which urine bags were used (urine collection kit: 2/68 [3%]; urine bag: 5/68 [7%]; *p* = 0.44). Novel urine collection kits using filter paper can collect samples from neonates safely and with a high probability of success.

## 1. Introduction

Urine provides important biological information for disease screening, diagnosis, and evaluation of the disease status. Therefore, urinalysis, which is non-invasive, has been frequently performed in general medical practice since ancient times. The methods for pediatric urine collection include clean-catch midstream urine, transurethral catheter insertion, suprapubic fine-needle aspiration, napkins, and urine bags [1,2,3]. Although there is a successful technique based on bladder stimulation and lumbar paravertebral massage maneuvers for collecting midstream clean-catch urine in newborns [4], midstream urine collection has not reached a general practice in neonates or infants as yet. Transurethral catheterization and suprapubic fine-needle aspiration are highly invasive procedures for pediatric patients. It has been reported that urinary phosphorus and calcium concentrations increase if the sample is collected using a napkin [3]. Therefore, in clinical practice, urine bags are often used to collect urine samples from neonates. This method has drawbacks such as contamination with stool and detachment of the bag due to body movements. In girls, urine collection might be difficult because of the inability to fix a urine bag successfully at the exact urethral meatus [5]. Dermatitis occasionally occurs with the use of tape fixation, and the possibility of contamination with skin bacteria is high [6]. Therefore, a method of urine collection that does not involve adhesion of the urine bag to the skin is desirable.

In 2019, Shino-Test Corp. (Tokyo, Japan) developed a qualitative urine collection kit containing ten filter papers of 3.2 mm diameter in a urine collection sheet (international patent application number: PCT/JP2020/044137). In this study, the usefulness and safety of the novel urine collection kit using this urine filter paper were clinically examined in neonates.

## 2. Materials and Methods

### 2.1. Subjects

A total of 141 neonates were admitted to Nihon University Itabashi Hospital between December 2019 and June 2020. Of them, 138 neonates were included; and three neonates were excluded, two of whom had insufficient records of clinical backgrounds and one for whom the urine collection sheet was not recovered. This observational study was approved by the Clinical Research Ethics Review Board of the Nihon University Itabashi Hospital (approval no. RK-191008-1; 18 October 2019), and written consent was obtained from the parents of the subjects.

### 2.2. Methods

**Figure 1:**
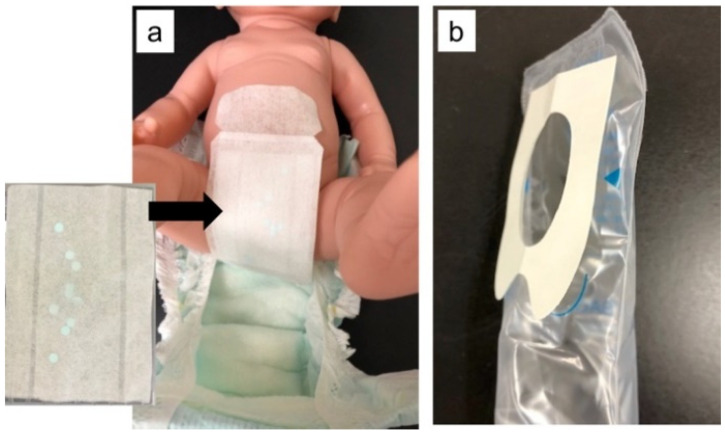
The novel urine collection kit (**a**) and conventional urine collection bag (**b**).

Clinical background data (sex, gestational age at birth, birth weight, birth height, and age on the urine collection date) of the neonates whose urine was collected were obtained. Urine was collected using a novel urine collection kit (Figure 1a). Urine sampling was performed during a single diaper change (approximately 3 h), regardless of whether urine had passed or not. The 68 neonates with the written consent for evaluating the presence of dermatitis (redness or rash) were evaluated after urine collection by the urine bag or novel urine collection kit.

**Figure 2:**
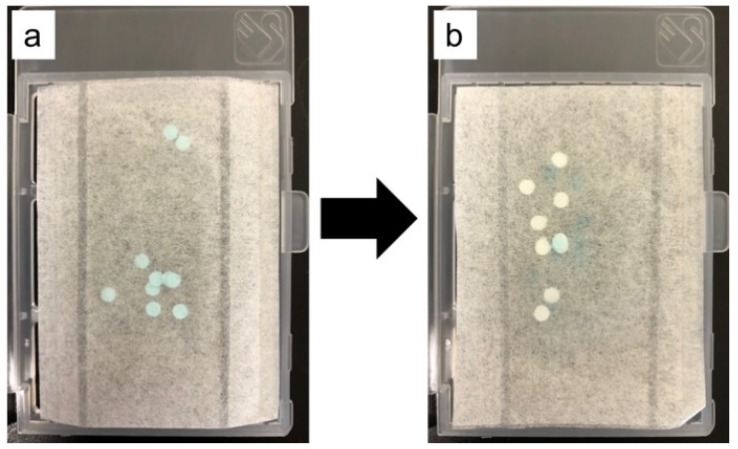
Changes in filter paper. (**a**) Dry filter paper (blue), (**b**) Wet filter paper discolored with urine (white).

The novel urine collection kit contained 10 filter papers with a 3.2 mm diameter in a urine collection sheet. The filter paper was coated with a water-soluble blue dye, and when water was applied, the blue dye turned white (international patent application number: PCT/JP2020/044137). The urine collection sheet was dried and stored in a specimen storage box. Among the 10 filter papers contained in the urine collection sheet, the number of filter papers whose color changed from blue to white was checked. Urine collection was considered to be successful when there was at least one filter paper with color change (Figure 2).

If the urine collection sheet was moist, urine was considered to have passed, and the urine collection rate was calculated based on the presence of any filter papers with color change. Effects of sex, age on the urine collection date, gestational age at birth, and birth weight on the urine collection rate were examined. Furthermore, we compared subjects with and without defecation at the time of urination. To confirm that the filter paper was wet because of urine, we detected creatinine from the filter paper sample. The incidence of dermatitis (redness or rash) was compared between when the novel urine collection kit was used and the urine bags were used (*n* = 68, Figure 1b).

### 2.3. Detection of Creatinine from the Filter Paper

**Figure 3:**
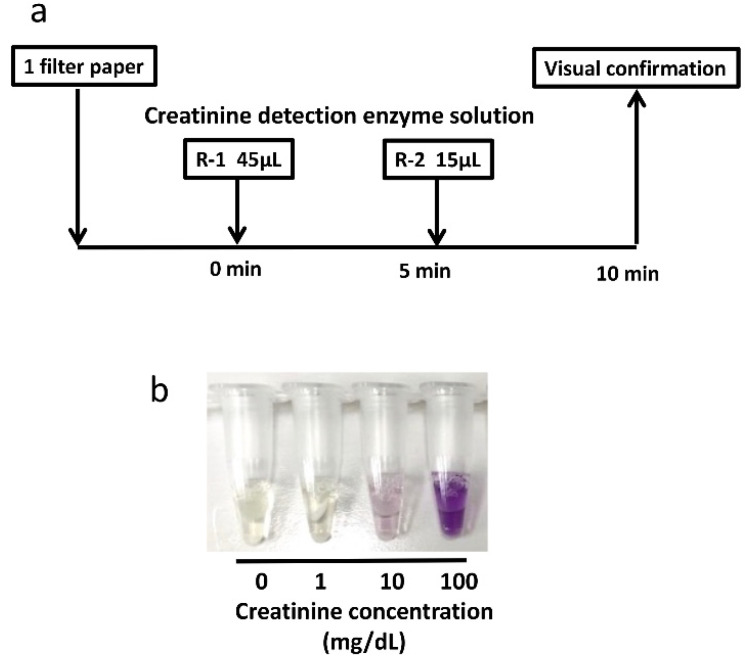
Method for detecting creatinine in a filter paper. (**a**): Schema of the method; (**b**): Color changes when creatinine is detected.

Cygnus auto-CRE (Shino-Test Corp., Tokyo, Japan) was used to measure urinary creatinine enzymatically. Schema of the method is shown in Figure 3: (1) Approximately 45 μL of R-I enzyme solution was added to one dried urine filter paper; (2) eight PCR tubes were heated in a heat block (set temperature 37 °C); (3) five minutes later, 15 µL of R-II enzymatic solution was added; and (4) changes in color were visually confirmed after 5 min. The R-I enzymatic solution contained creatinase, sarcosine oxidase, and N-ethyl-N-(2-hydroxy-3-sulfopropyl)-3-methoxyaniline sodium. The R-II enzymatic solution contained creatininase, 4-aminoantipyrine.

### 2.4. Statistical Analysis

Before the start of this study, no confirmatory statistical tests were performed because of the lack of previous papers for the novel urine collection kit; therefore, power calculation was not performed. For statistical analysis, JMP Pro 14 (SAS Institute Inc., Cary, NC, USA) was used, and the chi-square test, Fisher’s exact test, or Mann–Whitney U test were used appropriately. A significant difference was defined as *p* < 0.05.

## 3. Results

### 3.1. Subjects

Clinical backgrounds of the subjects are shown in Table 1. A total of 138 neonates including 42 preterm neonates were enrolled.

### 3.2. Urine Collection Rate

**Figure 4:**
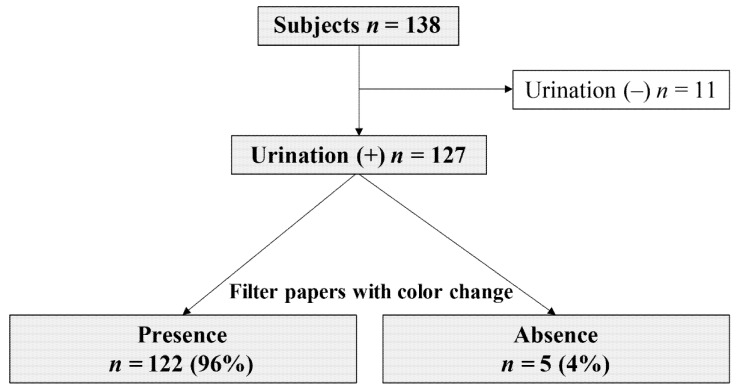
Flowchart for determining the urine collection rate with the novel urine collection kit.

Of the 138 neonates, 127 had urination during a single diaper change. Among them, the filter paper showed color changes in 122 neonates (urine collection rate: 96%; Figure 4). Of the 122 cases in which urine collection was successful, 98 (80%) showed changes in the color of all ten filter papers (Table 2).

### 3.3. Sex

Among the 127 neonates who had urination, 67 were boys and 60 were girls. Sex had no effect on the urine collection rate or number of filter papers with color changes (*p* = 1.00 and 0.84, respectively; Table 3).

### 3.4. Age at the Time of Urine Collection

Among the 127 neonates who had urination, 122 neonates whose age at the time of collection was known were included in the study. Urine was collected from 40 infants on the day of birth, 49 infants on day 1 after birth, 22 infants on day 2 after birth, and 11 infants after day 3 of birth. Age at urine collection had no effect on the urine collection rate or number of filter papers with color changes (*p* = 0.72 and 0.52, respectively; Table 4).

### 3.5. Gestational Age at Birth

The subjects were divided into two groups according to gestational age at birth: 40 in the preterm birth group and 87 in the term birth group. The gestational age at birth did not affect the urine collection rate or number of filter papers with color changes (*p* = 1.00 and 0.33, respectively; Table 5).

### 3.6. Birth Weight

The subjects were divided into three groups according to birth weight: 13 neonates weighing <1500 g, 34 weighing 1500 g to 2500 g, and 80 weighing ≥2500 g at birth. Birth weight had no effect on the urine collection rate or number of filter papers with color changes (*p* = 0.92 and 0.97, respectively; Table 6).

### 3.7. Stool Contamination

**Figure 5:**
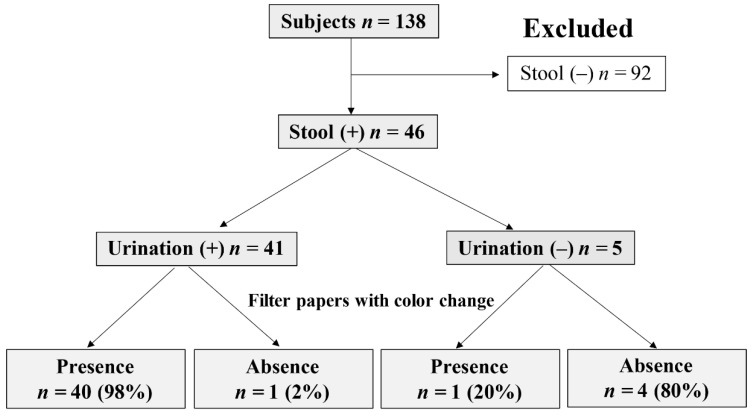
Flowchart for determining the urine collection rate in neonates with defecation.

Of the 138 subjects, 46 passed stools. Of them, we studied 41 neonates who had also urinated. The filter paper showed color changes in 40 neonates (urine collection rate: 98%), although a neonate who only defecated without urination had filter papers with color change (Figure 5). Even in the presence of stool, approximately 80% of the neonates showed color changes in 10/10 filter papers (Table 7). Stool contamination also had no effect on the number of filter papers with color changes compared to the group that only urinated (*p* = 1.00; Table 8).

### 3.8. Detection of Creatinine from the Filter Paper

**Figure 6:**
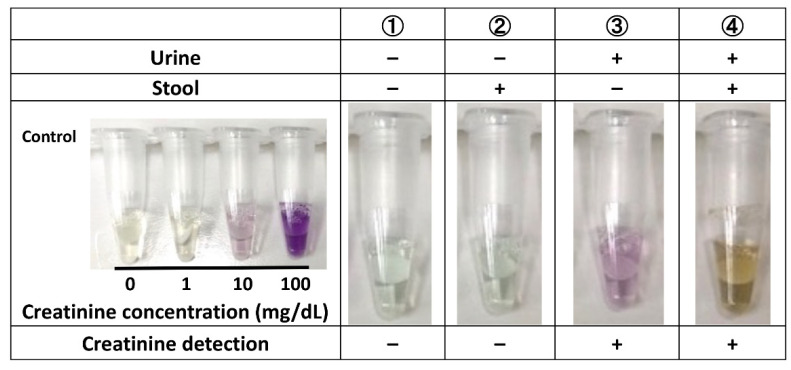
Detection of creatinine from the filter paper.

Creatinine was detected when the filter paper was moistened with urine, irrespective of the presence or absence of stool (Figure 6).

### 3.9. Incidence of Dermatitis Using the Urine Collection Kit or Urine Bag

With urine bags, dermatitis (redness or rash) was identified in five (7%) out of 68 neonates. The novel urine collection kit identified dermatitis in two out of 68 (3%) neonates. The use of urine collection sheets did not lead to an increase in dermatitis compared to that with the use of urine bags (*p* = 0.44; Table 9).

## 4. Discussion

This study showed that the urine collection rate with the novel urine collection kit using urine filter papers was high (96%). Sex, birth weight, or gestational age had no impact on the urine collection rate. Moreover, its use did not increase the number of neonates with dermatitis compared to the use of urine bags. In our small cohort study, the novel urine collection kit showed no safety concerns compared to urine bags. It also minimized contamination with stool with an outer protective sheet.

Because the novel urine collection kit cannot be used to obtain the urine volume, it is suitable for screening biochemical substances and human or viral genomic DNA in urine. Examples of clinical applications that we considered are as follows: semi-quantitative measurements of urinary creatine and creatinine for cerebral creatine deficiency syndrome [7]; the semi-quantitative measurement of urinary β2-microglobulin for congenital anomalies of the kidney and urinary tract [8,9]; mutation analyses of type VI collagen and the α5 gene for Alport syndrome using genomic DNA isolated from urinary sediments [10]; and detection of urinary cytomegalovirus DNA for congenital cytomegalovirus infection because it can be a diagnosis of the detection of urinary cytomegalovirus DNA in neonates ≤3 weeks of age [11,12].

Although the method of urine sampling with cotton placed in the diaper and the urine seeping in it has been used before [3,13,14], contamination with stool, stool-derived leukocytes, bacteria and constituents, and skin bacteria on the external vulva is a concern, particularly in girls [3,15]. Biochemical urinalysis using cotton showed high levels of phosphorus and calcium [3]. Currently, urine bags are used for children as the main method of urine sampling.

Urine bags are useful for biochemical examination, which requires a large volume of urine collection. However, neonates weighing <1500 g can often develop vulval inflammation from tape fixation because of their immature skin, and girls are likely to have leakage from the urine bag [16]. Furthermore, there is considerable contamination of the skin bacteria, and urine samples are unsuitable for the diagnosis of urinary tract infections [6,17,18]. A Brazilian study using pads reported that the rate of contamination of urine with bacteria was similar to the use of pads and urine bags and was significantly higher compared to clean-catch midstream urine [19]. Pyuria in bag-collected urine specimens might consist of leukocytes from the external genitalia and urinary tract [20]. Since urine collected in absorbable pads is usually contaminated, the novel urine collection kit using urine filter paper in this study is not useful for the diagnosis of urinary tract infections. However, it is possible to execute some urinalyses. Some researchers have reported that a collection of urine from disposable nappies that are not soiled by feces is an inexpensive, rapid, and simple method for the culture for bacteria and biochemical analysis such as nitrites [21,22]. Further studies are needed to validate if the novel urine collection kit using urine filter paper can be used for these urinalyses.

As a limitation of this study, we did not examine whether creatinine was detected in all wet filter papers. However, creatinine would be detected in all filter papers with color change because the color does not change without enough water such as urine in a diaper.

## 5. Conclusions

With the novel urine collection kit, the urine collection rate was 96%. When urine could be collected, 80% of the subjects showed color change in 10/10 filter papers. Sex, age at collection, preterm birth, birth weight, or fecal contamination did not affect the urine collection rate. There was no increase in the incidence of dermatitis compared to the use of urine bags.

## 6. Patents

A novel urine collection kit using filter paper was applied to the International Patent Office on 28 November 2019, by Shino-Test Corp. (Tokyo, Japan; international application number: PCT/JP2020/044137).

## Figures and Tables

**Table 1:** Clinical background of the subjects.

	*n* = 138
Male, *n*	72 (52)
Gestational age, weeks	38 (26–41)
Birth weight, g	2799 (464–3804)
Birth height, cm	47.7 (28.4–52.5)
Age on the collection date, days	1 (0–5)
Preterm newborns (<37 weeks of gestational age at birth), *n*	42 (30)
Newborns <1500 g of birth weight at birth, *n*	13 (9)

Data are shown as median (range) or number (percentage).

**Table 2:** Number of filter papers with color change.

*n* = 122	1–5	6–9	10
Neonates with urine collection	11 (9)	13 (11)	98 (80)

Data are shown as number (percentage).

**Table 3:** Comparison of the urine collection rate between the sexes.

	Boys, *n* = 67	Girls, *n* = 60	*p*-Value
Urine collection rate	64 (96)	58 (97)	1.00 *
Number of filter papers with color change	10 (0–10)	10 (0–10)	0.84 **

Data are shown as median (range) or number (percentage). *p*-values were calculated using Fisher’s exact test * or Mann–Whitney U test **.

**Table 4:** Comparison of the urine collection rate by age at the time of collection.

	0 day *n* = 40	1 day *n* = 49	2 days *n* = 22	≥3 days *n* = 11	*p*-Value
Urine collection rate	39 (96)	45 (97)	22 (100)	11 (100)	0.72 *
Number of filter papers with color change	10 (0–10)	10 (0–10)	10 (1–10)	10 (2–10)	0.52 **

Data are shown as median (range) or number (percentage). *p*-values were calculated using the chi-square test * or Mann–Whitney U test **.

**Table 5:** Comparison of the urine collection rate between preterm and term neonates.

	Preterm, *n* = 40	Term, *n* = 87	*p*-Value
Gestational age, weeks	34 (26–36)	38 (37–41)	<0.0001 **
Urine collection rate	39 (98)	83 (95)	1.00 *
Number of filter papers with color change	10 (0–10)	10 (0–10)	0.33 **

Data are shown as median (range) or number (percentage). *p*-values were calculated using Fisher’s exact test * or Mann–Whitney U test **. Preterm, <37 weeks of gestational age at birth; Term, ≥37 weeks of gestational age at birth.

**Table 6:** Comparison of the urine collection rate among newborns classified by birth weight.

	<1500 g *n* = 13	1500–2499 g *n* = 34	≥2500 g *n* = 80	*p*-Value
Birth weight, g	1146 (464–1424)	2146 (1520–2495)	3014 (2512–3804)	<0.0001 **
Urine collection rate	12 (92)	32 (94)	78 (98)	0.92 *
Number of filter papers with color change	10 (0–10)	10 (0–10)	10 (0–10)	0.97 *

Data are shown as median (range) or number (percentage). *p*-values were calculated using the chi-square test * or Mann–Whitney U test **.

**Table 7:** Number of filter papers with color change in neonates who urinated and defecated.

	0	1–5	6–9	10
Neonates who urinated and defecated, *n* = 41	1 (2)	4 (10)	5 (12)	31 (76)

Data are shown as number (percentage).

**Table 8:** Comparison of urine collection in neonates between those who urinated and defecated and those who only urinated.

	0	1–5	6–9	10	*p*-Value
Urinated and defecated, *n* = 41	1 (2)	4 (10)	5 (12)	31 (76)	1.00
Only urinated, *n* = 86	4 (5)	7 (8)	8 (9)	67 (78)	

Data are shown as number (percentage). The *p*-value was calculated using the chi-square test.

**Table 9:** Comparison of the incidence of dermatitis between the two methods.

Urine Collection Method, *n* = 68	Dermatitis (+)	Dermatitis (−)	*p*-Value
Urine bag	5 (7)	63 (93)	0.44
Novel urine collection kit	2 (3)	66 (97)	

Data are presented as number (percentage). The *p*-value was calculated using Fisher’s exact test.

## Data Availability

The data presented in this study are available on request from the corresponding author.
